# Epidemiological Insights into Endoparasites of Brown Bears (*Ursus arctos*) in Greece

**DOI:** 10.3390/pathogens15070671

**Published:** 2026-06-25

**Authors:** Antonios Synapalos, Anastasia Diakou, Stefanos Sgardelis

**Affiliations:** 1 Department of Ecology, School of Biology, Aristotle University of Thessaloniki, 54124 Thessaloniki, Greece; asynapalo@bio.auth.gr; 2 School of Veterinary Medicine, Faculty of Health Sciences, Aristotle University of Thessaloniki, 54124 Thessaloniki, Greece

**Keywords:** *Baylisascaris transfuga*, brown bear, endoparasites, epidemiology, Greece, seasonality, spatio-temporal

## Abstract

Brown bear populations in Greece face multiple threats, and parasitic infections may pose an additional risk to these vulnerable animals. This study represents the first comprehensive assessment of endoparasite occurrence, prevalence, and seasonality in brown bears in Greece, in relation to geographical location and the animal’s different physiological phases. A total of 918 faecal samples were collected over a three-year period from regions with brown bear presence in Greece. For each sample, the date of collection and the coordinates of the site were recorded. Samples were examined using sedimentation, flotation, and McMaster techniques, while the Baermann method was additionally applied to a subset of 195 samples. Spatial and temporal patterns in parasite occurrence and diversity were analysed using generalised additive models (GAMs). Ten parasitic taxa were identified, with *Baylisascaris transfuga* being the most prevalent (39.8%), followed by *Crenosoma* spp. (26%), *Uncinaria* spp. (18.09%), and *Dicrocoelium dendriticum* (14.38%). Less prevalent taxa included *Eucoleus aerophilus*, *Sarcocystis* spp., *Toxascaris leonina*, *Eimeria* spp., *Linguatula serrata*, and Taeniidae. Μixed infections, involving two or more parasites, were detected in 22% of the samples. The prevalence of *B. transfuga* was higher in late autumn, with high-risk infection areas identified in both late summer and autumn. In contrast, *Uncinaria* spp. and *D. dendriticum* showed no seasonal variation, while *D. dendriticum* exhibited spatial clustering patterns similar to *B. transfuga* but without clear seasonal trends. These findings highlight the widespread occurrence and complexity of parasitic infections in Greek brown bears. Continued long-term monitoring is essential to improve understanding of transmission dynamics and the ecological processes shaping parasite distribution in this animal species.

## 1. Introduction

The brown bear (*Ursus arctos*) population in Greece shows promising signs of recovery over the last 15 years [1,2]. The species is distributed in two distinct subpopulations located in northwestern (Pindos-Peristeri mountain range) and northeastern Greece (Rhodope mountain complex) [3] with approximately 500 individuals [2,4]. However, bears in Greece still face significant challenges, including deforestation and poaching, which have been characterised as “high-intensity” threats. Other challenges, such as infrastructure (roads, dams, wind farms), poisoned baits, and climate change, are categorised as medium-intensity threats [5]. 

Although human activities and environmental deterioration pose the major threats to wildlife, under certain conditions, infectious diseases may also adversely affect small or isolated populations [6]. Among the agents of infectious diseases, parasites are generally understudied but can impair the health status and overall fitness of bears. Furthermore, climate change, land-use change, and food scarcity in natural habitats force animals to move closer to human settlements. Consequently, bears, domestic animals, and humans share sympatric zones that serve as a terrain of possible bridging infections [7,8].

People and wildlife in Europe coexist in an anthropogenically modified landscape that favours the circulation of pathogens among wild animals, domestic animals, and people [1,8]. Therefore, there is merit in investigating brown bear parasites and assessing their effect and importance on this animal species, domestic animals, and public health [9,10].

Studies on brown bear endoparasites in Europe are scarce, and information about their impact is limited. Most of the recent studies come from Slovakia [11,12], Croatia [13], Romania [14], Estonia [15], Spain [16,17,18], and the East Carpathians Biosphere Reserve (Slovakia/Poland/Ukraine) [19]. In Greece, two case reports have been published, reporting two parasites, i.e., *Dirofilaria immitis* and *Thelazia callipaeda*, which are common in carnivores and other mammals [20,21]. The aim of the present study was to investigate, for the first time, the endoparasite fauna of brown bears in Greece and to examine the seasonality of parasitism, in relation to geographical location and the animal’s different physiological phases throughout the year.

## 2. Materials and Methods

### 2.1. Study Area and Sampling Sites

**Figure 1:**
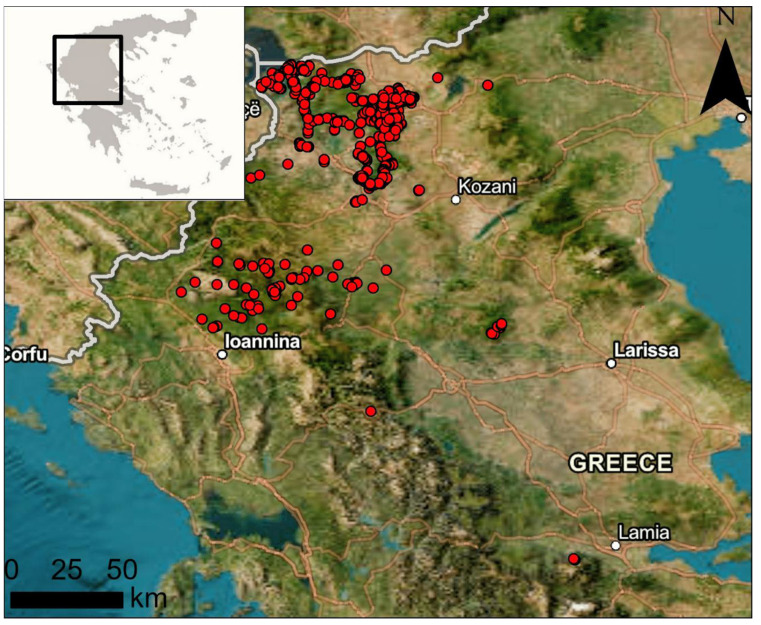
Study area and brown bear faecal sampling sites (red dots). The upper cluster of sites corresponds to Western Macedonia (Prespes National Park) and the lower to Northern Pindos (Northern Pindos National Park).

Sampling took place mostly in regions with national parks, i.e., Western Macedonia (Prespes National Park) and Northern Pindos (Northern Pindos National Park), as well as a few additional locations with documented brown bear presence in Greece (Figure 1). All regions have a continental Mediterranean climate, with cold winters and mild summers, and altitudes ranging from 400 m to 2524 m. Throughout the study area, there are mountain ranges, lakes, rivers, semi-mountainous areas, hills, and lowlands. Forests are mainly broadleaf, coniferous, or a combination of the above [22]. Four major cities (Kozani, Florina, Grevena, and Kastoria) and numerous towns and villages create a mosaic of urban, rural, and natural landscapes. Animal husbandry and various forms of agriculture (e.g., crops, orchards, vineyards) constitute the main rural activities in the area. Sampling sites were chosen based on telemetry data [22] (Figure 1).

### 2.2. Sampling

**Figure 2:**
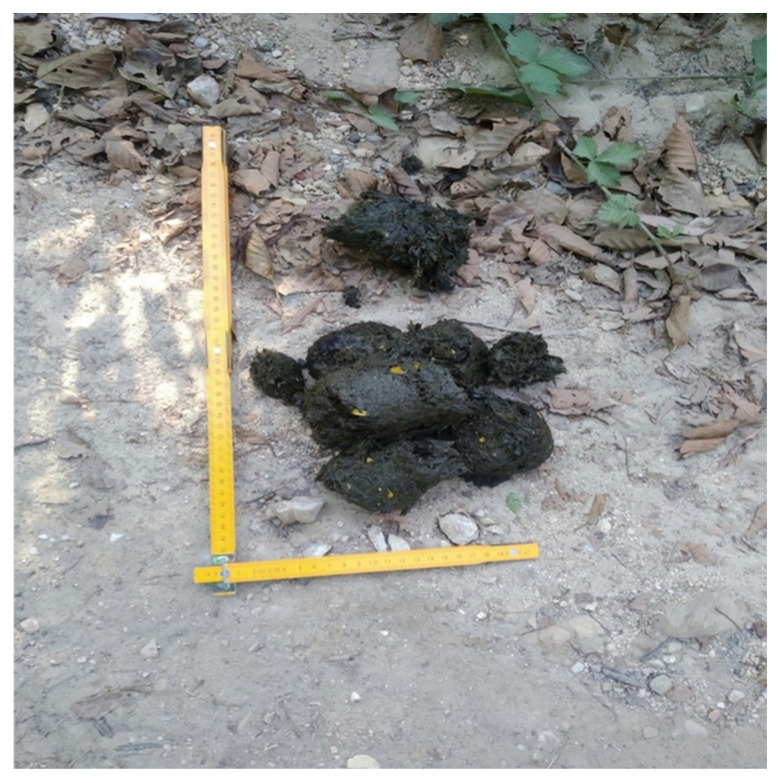
Brown bear faecal sample before collection.

Brown bear faecal samples were collected during 5-day sampling trips conducted on a monthly to bimonthly basis during 2020–2022 and 2024, mainly along forest roads, around feeding sites, and in areas of bear activity (e.g., abandoned orchards, corn fields). Overall, 918 brown bear faecal samples, identified by their morphological characteristics (size, shape, visible food components), were collected [23]. Each sample was photographed (Figure 2) and then placed in a single-use, airtight plastic bag. To limit sampling bias caused by sampling the same individual multiple times, each site was visited only once during each sampling trip, and the sites were several kilometres apart. 

Each plastic bag was tagged with a unique code. For each sample, location, GPS coordinates (Garmin Instinct Solar watch, New Taipei City, Taiwan), and collection date were recorded. The samples were stored at 4 °C until examination.

### 2.3. Parasitological Examinations

All faecal samples were transferred to the Laboratory of Parasitology and Parasitic Diseases, School of Veterinary Medicine, Aristotle University of Thessaloniki, and examined within 3−7 days of collection. All samples were examined by two standard parasitological methods: (i) ZnSO_4_ flotation with centrifugation [24] and Telemann (formaldehyde, ether) sedimentation [25] for the detection of parasitic elements. Samples deemed sufficiently fresh (i.e., retaining their shape and moisture and showing no visible fungal growth) were also examined using the Baermann method to detect first-stage larvae (L1) [24]. The preparations were examined under the microscope (100×, 400×), and the parasitic elements were identified based on their morphological and morphometric characteristics to the lowest taxon possible, i.e., species, genus, or family, depending on the presence of distinguishing morphological traits [26,27]. Positive samples were further analysed by the modified McMaster method [28] to quantify the parasite eggs per gram of faeces (EPG).

### 2.4. Statistical Analysis

Spatial and temporal patterns in parasite occurrence and diversity were analysed using generalised additive models (GAMs), selected for their ability to flexibly model non-linear relationships and spatial structure. Specifically, we used a tensor-product smooth of sample coordinates to model spatial clustering of infection, thus accounting for possible spatial autocorrelation of the data. Sample coordinates were available for a subset of 859 samples out of the original 918, and this subset was used for modelling.

Presence–absence responses were modelled using a binomial error distribution with a logit link. All models included spatial location, represented by longitude and latitude, and temporal parameters, i.e., season and year of study. To examine the seasonality of parasitological findings, seasons based on bear activities, as defined by de Gabriel Hernando et al., 2015 [2], were used. Seasons were defined as emergence (EM), 1 March–21 April; mating (MA), 22 April–7 August; early hyperphagia (EH), 8 August–7 October; and late hyperphagia (LH), 8 October–15 December. To allow spatial patterns to differ among seasons, spatial smooths were fitted using season-specific smooth terms.

The model selection was guided by the Unbiased Risk Estimator (UBRE) score of the binomial GAM. All models were fitted using Restricted Maximum Likelihood (REML). Spatial terms were interpreted as evidence of geographic clustering when clear spatial structure was present. The model fit was assessed using the proportion of deviance explained and the adjusted R^2^ values and Area Under the Curve (AUC).

Predicted spatial patterns were visualised using contour plots based on model predictions on a regular spatial grid. For presence–absence models, points indicating observed parasite presence were overlaid to illustrate the correspondence between predicted high-risk infection areas (“hotspots”) and observed infections. Only seasons exhibiting clear spatial structure are presented in the results herein.

All analyses were conducted in R (version 4.5.0) [29], primarily using the mgcv package [30] for model fitting, oddsratio [31] for the calculation of odds ratios for GAMs, DHARMa [32] for residual diagnostics based on 1000 simulations, pROC [33] for AUC estimation, and lattice/grid graphics for visualisation [34].

## 3. Results

### 3.1. Intestine Parasite Taxa and Their Prevalence

**Figure 3:**
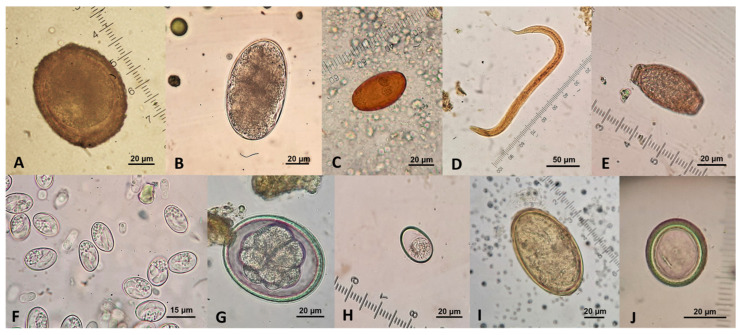
Diagnostic stages of parasites found in brown bear faecal examinations in Greece. (**A**) *Baylisascaris transfuga* egg; (**B**) *Uncinaria* spp. egg; (**C**) *Dicrocoelium dendriticum* egg; (**D**) Lugol-stained *Crenosoma* spp. L1; (**E**) *Eucoleus aerophilus* egg; (**F**) *Sarcocystis* spp. sporocysts; (**G**) *Toxascaris leonina* egg; (**H**) *Eimeria* spp. oocyst; (**I**) *Linguatulla serrata* egg; (**J**) Taeniidae egg.

Of the 918 faecal samples,134 were collected in EM, 192 in MA, 294 in EH, and 298 in LH. Overall, 514 (56%) were positive for parasitic elements, i.e., eggs, oocysts, sporocysts, or larvae. Ten different parasitic taxa were found, of which three, i.e., *Baylisascaris transfuga*, *Uncinaria* spp., and *Dicrocoelium dendriticum,* were the most common, with prevalences of 39.98%, 18.09%, and 14.38%, respectively. The remaining seven taxa showed a prevalence of 2% or less. The prevalence of *Crenosoma* spp. (26.05%) was determined based on Baermann method results from 195 samples (Table 1, Figure 3).

The McMaster method yielded a positive result (≥50 EPG) only for *B. transfuga* eggs (detailed results in Section 3.2).

### 3.2. Spatio-Temporal Variation in Prevalence

**Figure 4:**
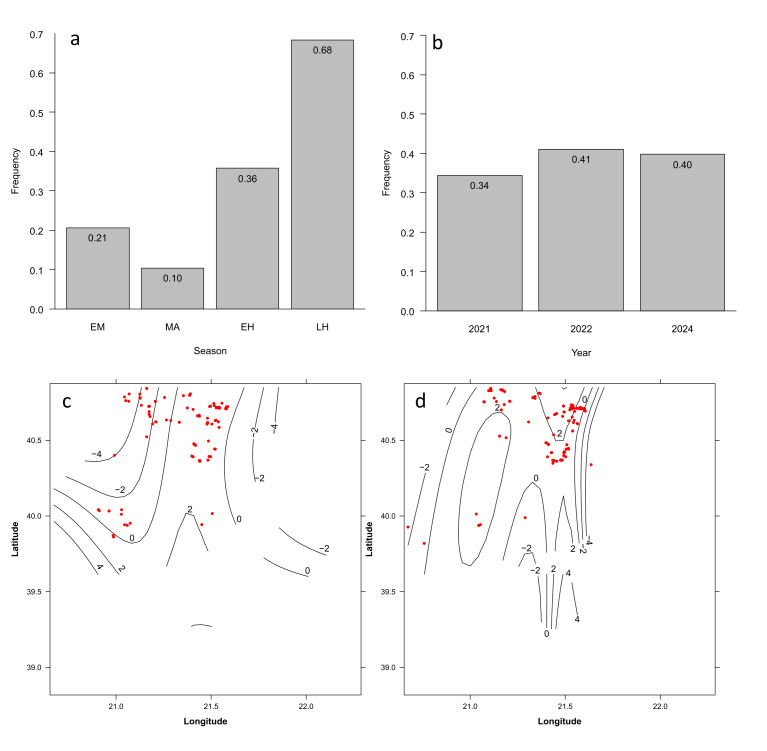
*Baylisascaris transfuga* prevalence in brown bears in Greece per season (**a**) and per year of study (**b**). Spatial structure during EH (**c**) and LH (**d**). EM: emergence season, MA: mating season, EH: early hyperphagia season, LH: late hyperphagia season. Panels (**c**,**d**): red points denote observed positive samples. Contour lines represent centred partial effects. Zero-level contours represent the average model prediction. Areas enclosed by higher contour levels (positive effect) correspond to regions of greater predicted infection.

Prevalence of *B. transfuga* varied strongly with both season and year (Figure 3A,B, Table 2). Its overall prevalence was lower during MA compared to EM and significantly higher during LH. No significant difference was detected for EH relative to EM (*p* = 0.58). Prevalence was significantly higher in 2022 and 2024 compared to 2021 (Figure 4b, Table 2).

Spatial clustering of positive samples was season-dependent (Figure 3C,D and Table 2), namely, a limited spatial structure was observed during EM and MA, whereas clear and persistent geographic hotspots emerged during EH (edf = 11.0, *p* = 0.023) and LH (edf = 5.6, *p* = 0.022) (Figure 3C,D). The model explains 25.3% of the total deviance with an adjusted R^2^ of 0.267 and AUC = 0.83.

**Figure 5:**
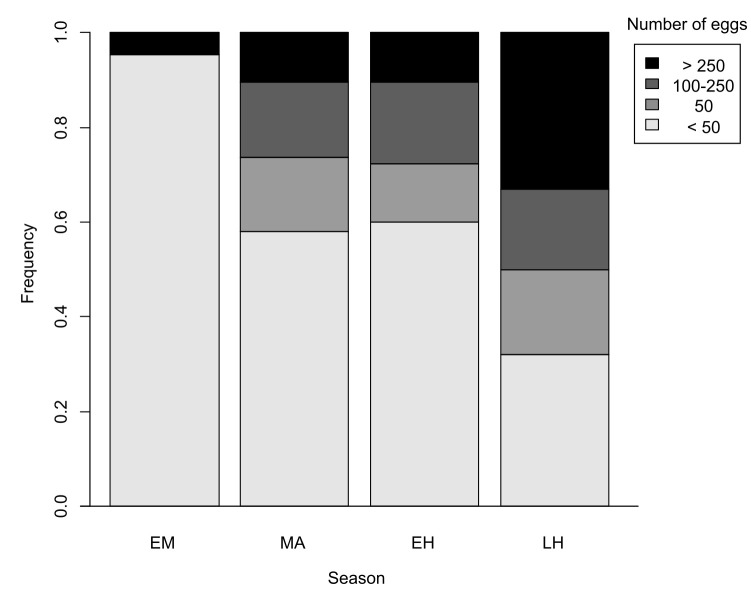
Frequency of *Baylisascaris transfuga* eggs per gram (EPG) classes per season. The first EPG class (<50) includes samples with egg counts per gram below the McMaster method’s detection threshold. EM: emergence season, MA: mating season, EH: early hyperphagia season, LH: late hyperphagia season.

Infection intensity (EPG) was estimated in samples positive for *B. transfuga*. The EPG values ranged from 50 to 2000 with a median of 150. In EH the infection intensity was low. Most positive samples had fewer than 50 EPG (below the McMaster method’s detection threshold), while a few samples had more than 250 EPG. MA and EH showed similar distributions of infection intensity with an almost even representation of the three classes with more than 50 EGP and a slightly higher representation of samples with less than 50 EPG. Finally, the LH distribution shows a shift towards samples with >250 EPG (Figure 5).

The prevalence of *Uncinaria* spp. infection in different seasons varied between 15.6 % in MA and 21.5% in LH, but this variation was not statistically significant (Table 3). There was a tendency for the prevalence to decrease over the years. Furthermore, no significant spatial clustering of *Uncinaria* spp. positive samples were observed. The model explained 11.9% of the total deviance with an adjusted R^2^ of 0.07 and AUC = 0.7.

**Figure 6:**
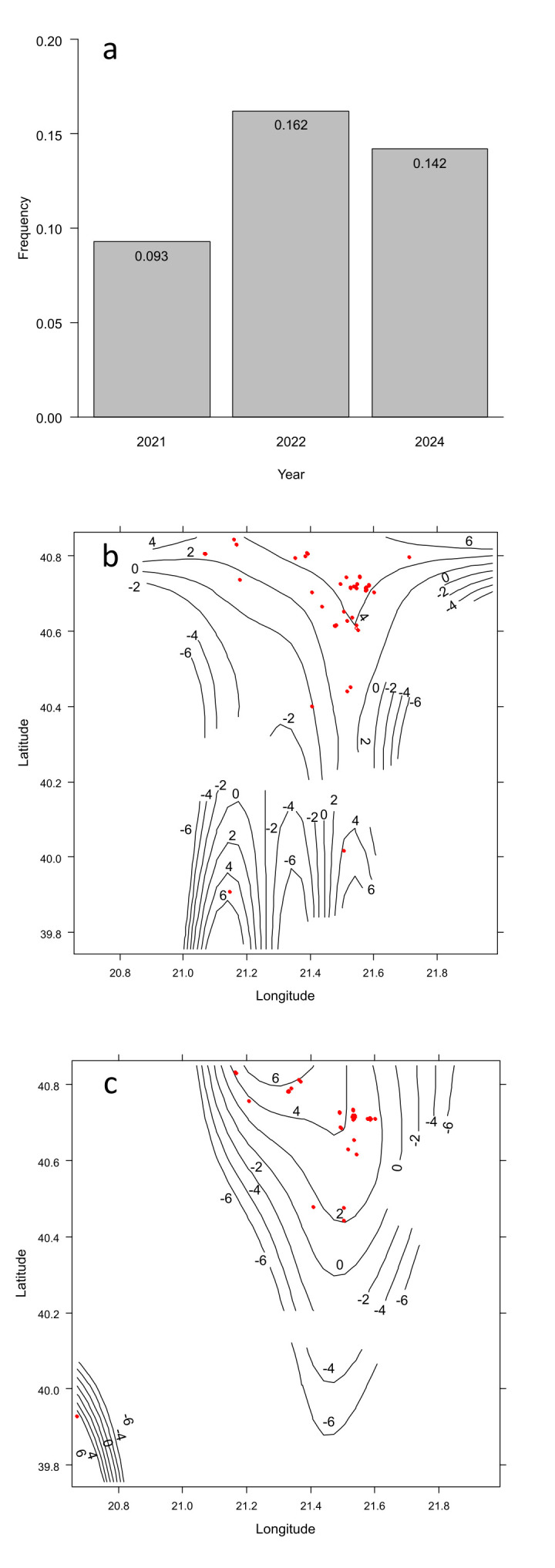
*Dicrocoelium dendriticum* prevalence per year of study (**a**) and spatial structure during EH (**b**) and LH (**c**) seasons. EM: emergence season, MA: mating season, EH: early hyperphagia season, LH: late hyperphagia season. Panels (**b**,**c**): red points denote observed positive samples. Contour lines represent centred partial effects. Zero-level contours represent the average model prediction. Areas enclosed by higher contour levels (positive effect) correspond to regions of greater predicted infection.

Similarly to *Uncinaria* spp., *D. dendriticum* showed no statistically significant seasonal variation, showing a minimum during MA (9.4%) and a maximum during LH (16.8 %). However, there was a significant variation on a year-by-year basis, with higher prevalence during 2022 and 2024 compared to 2021 (Figure 5 and Table 4). As in the case of *B. transfuga,* there was a significant spatial clustering of *D. dendriticum*-positive samples during both EH and LH (Figure 6). The model explained 21.1% of the total deviance with an adjusted R^2^ of 0.14, AUC = 0.79. 

Finally, no statistical analysis was performed for *Ε. aerophilus*, *Sarcocystis* spp., *T. leonina*, *Eimeria* spp., *L. serrata*, and Taeniidae due to their low prevalence, and in the case of *Crenosoma* spp. due to the selective sampling (only fresh samples) that could bias any statistical result.

### 3.3. Mixed Infections

**Figure 7:**
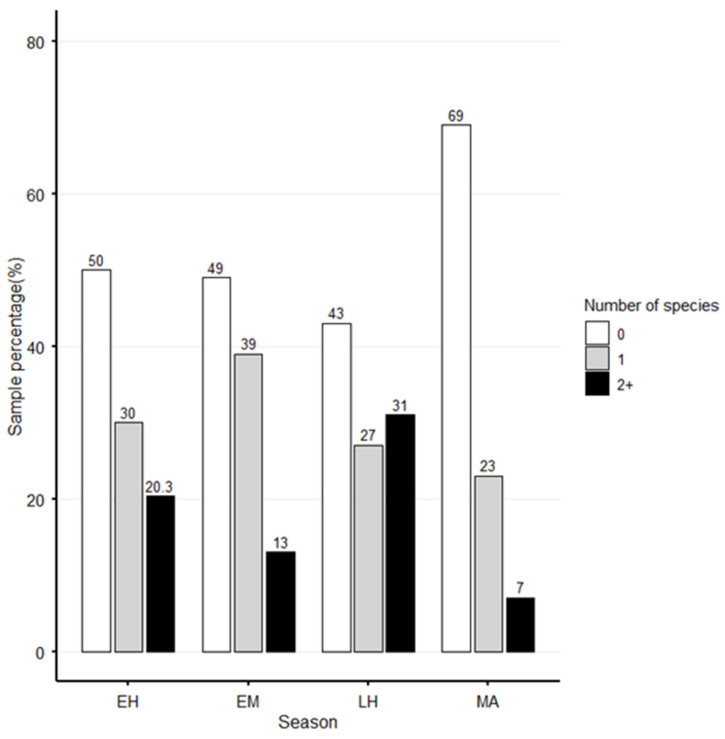
Percentage of faecal samples with 0–2+ parasite species per season. EM: emergence season, MA: mating season, EH: early hyperphagia season, LH: late hyperphagia season.

Mixed infections with two or more parasite taxa were recorded in 194 (22%) samples (Figure 7). The most common co-infection documented was *B. transfuga* with *Uncinaria* spp. (41%), followed by *B. transfuga* with *D. dendriticum* (26%), and *B. transfuga* with *Crenosoma* spp. (16%). Mixed infections with more than two different parasite taxa were less prevalent.

## 4. Discussion

This study presents the first systematic investigation of endoparasites in brown bears and their relationship to the species’ ecology in Greece. A total of ten parasitic taxa were identified from faecal samples, including protozoa, trematodes, cestodes, nematodes, and pentastomids.

Morphological identification of brown bear scat is a highly reliable method due to the absence of other bear species on the continent [23,35]. Although substantial effort was undertaken to reduce the probability of repeatedly sampling the same individual during a single sampling trip, faecal samples were collected opportunistically from the environment without genetic identification of individuals. As a result, repeated sampling of some bears cannot be excluded. Such pseudoreplication may have introduced bias into the baseline prevalence estimates. Moreover, because these observations were subsequently used in the predictive modelling framework, the same bias may have propagated into the model predictions.

Morphological identification to the species level was possible for five parasites, i.e., *B. transfuga*, *D. dendriticum*, *E. aerophilus*, *T. leonina*, and *L. serrata*, due to the characteristics of their eggs that distinguish them from similar species. These characteristics include the shape and size of the eggs, the morphology of the eggshell (e.g., thickness, surface texture, presence/absence of operculum), and the nature and morphology of the egg content (e.g., zygote, larva, or miracidium) [25,36,37]. On the other hand, the genera *Uncinaria*, *Crenosoma*, *Sarcocystis,* and *Eimeria* include different species that share common morphological characteristics in their diagnostic stages (eggs, larvae, sporocysts, and oocysts, respectively); thus, identification was restricted to the genus level [27,38]. Finally, most of the species included in the family Taenidae have morphologically similar eggs; thus, these eggs were identified to the family level [25].

Ascarid nematodes of the genera *Baylisascaris* and *Toxascaris* were recorded in the samples examined. *Baylisascaris* was the most prevalent parasite found. Although molecular identification of the species was not performed, the most likely species involved is *B. transfuga*, a parasite of ursids documented in all eight bear species [39]. Previous studies in Europe have reported varying prevalences, with higher rates in Slovakia (52.9%) [40], Estonia (51%) [15], and Spain (44.8%) [18], whereas in Croatia, Romania, and Italy the prevalences were lower (<20%) [13,14,41]. The variation in prevalence observed across these studies may be attributed to differences in sampling season, with a consistent finding of higher *B. transfuga* prevalence during autumn [15,17,18,19]. Though rare, clinical manifestations of baylisascariosis can include anorexia, poor coat condition, and intestinal obstruction [42]. While *B. transfuga* has not been confirmed to infect non-ursid species in Europe [10], all members of the genus are considered potentially zoonotic and may cause visceral, ocular, or neural larva migrans in humans [40]. The parasite’s life cycle is not fully described, but it is most likely similar to that of other ascarids, with infection occurring through the ingestion of embryonated eggs or paratenic hosts [14]. *Toxascaris leonina* is a common roundworm reported in domestic and wild canids and felids all around the world [43]. Eggs morphologically compatible with *T. leonina* have been reported in bears before [44,45]. Bears are not a confirmed host of this parasite, and the scenario of pseudoparasitism, i.e., eggs appearing in bears’ faeces as a result of consuming an infected carnivore, cannot be ruled out [42].

Eggs of the family Ancylostomatidae (hookworms) have been recorded in bears from Europe [12,13,35] and South America [46]. Within this family, two species of the genus *Uncinaria* have been identified in North America in American black bears (*Ursus americanus*) and grizzly bears (*Ursus horribilis*), i.e., *Uncinaria yukonensis* and *Uncinaria rauschi* [47,48]. The species *Uncinaria stenocephala* has been identified in brown bears in Russia [49]. Finally, another species, *Uncinaria ursi,* has been found in polar bears [50]. Recent genetic studies in North America suggest a close relationship between these *Uncinaria* species and hypothesise similar infection patterns [51]. Namely, the third-stage larvae from the environment enter a suitable host by skin penetration or oral ingestion and complete their life cycle in the host’s small intestine [52]. In Greece, *Uncinaria* spp. has been detected in both wild and domestic carnivores that share the same habitat as brown bears [20,53]. In the present study, the parasite shows the second-highest prevalence among bear gastrointestinal parasites (19%), which agrees with findings from Croatia (10%) [13]. A higher prevalence of Ancylostomatidae has been recorded in Slovakia (33%) and Italy (51%) [12,41]. Genetic research is needed to identify the species of *Uncinaria* in European brown bears and to determine its impact on the health of domestic and wild carnivores.

The trematode *D. dendriticum* was found here at a prevalence of 14%, while prevalences in recent studies in Europe vary widely, from 0.4% in Romania [14] to very high percentages in the Cantabrian Mountains, Spain (32–71%) [16,17,18]. Adult *D. dendriticum* resides in the bile ducts and gall bladder of its definitive hosts, causing lesions in the liver and, in severe cases, hepatitis and cirrhosis. The parasite’s eggs are shed in the faeces of the definitive host, and its life cycle involves two intermediate hosts: terrestrial gastropods as the first and ants as the second intermediate host [53]. Bears consume ants as part of their natural diet [54], which may lead to infection by this parasite. However, it cannot be ruled out that the detection of *D. dendriticum* eggs in some cases may be due to pseudoparasitism resulting from the consumption of infected definitive hosts’ (e.g., ruminants or lagomorphs) liver tissue.

Two nematodes affecting the respiratory system were found: *Eucoleus aerophilus* and *Crenosoma* spp. *Eucoleus aerophilus* (syn. *Capillaria aerophila*) has been reported in American black bears in the Southeastern United States [55] in a relatively high prevalence (10%). The presence of *Eucoleus* has been documented in brown bears in Slovakia [12,56], Italy, and Russia [41,57]. In Greece, the parasite has been detected in European wildcats and domestic cats [58,59]. This nematode has a direct life cycle and resides in the lungs (bronchi and trachea) of the hosts. The female releases eggs, which are transported by the mucociliary escalator to the pharynx, where they are swallowed and excreted in faeces. New hosts subsequently become infected by ingesting larvated eggs or, potentially, infected earthworms [60,61,62]. Clinical symptoms include dry and productive cough, sneezing, distress, dyspnea, and trachypnea, and range from mild to severe [62,63]. However, there is no evidence of clinical capilariosis in wild bears.

The diagnostic stage of *Crenosoma* spp. in the host’s faeces is the L1, which is less resistant to dryness than eggs and oocysts. Therefore, to obtain results closer to the actual prevalence of this parasite, only samples deemed sufficiently fresh were submitted to the specific larval retrieval method (Baermann). *Crenosoma* spp. have been previously reported in both American black bears [49,55,64] and brown bears in Romania [14], with lower prevalence than that recorded in the present study (5.45%). However, the gold-standard method for larval detection, i.e., the Baermann method [24], was not used in those studies, suggesting that the true prevalence of the parasite in those areas may be higher. Other animal species, such as wild and domestic canids, are also susceptible to *Crenosoma* spp. infections [65,66] and can act as reservoirs. The parasite uses gastropods as intermediate hosts, while several small vertebrates (e.g., amphibians, rodents, and reptiles) can act as paratenic hosts [67]. Adult parasites reside in the bronchi and can cause cough, bronchitis, and, in severe cases, pneumonia [68,69]. There is one clinical case in bears involving an orphaned black bear cub with mild symptoms [70]. It is unclear whether adult bears in the wild develop symptoms due to crenosomosis. 

Protozoan parasites had a low prevalence in the present study, with only two genera, i.e., *Sarcocystis* spp. (0.5%) and *Eimeria* spp. (0.4%), recorded. *Sarcocystis* spp. have an indirect life cycle: definitive hosts acquire the infection by consuming intermediate hosts bearing tissue “sarcocysts”. In the definitive host, the parasite multiplies in the enterocytes and is shed into the environment via faeces, from which the intermediate host becomes infected [71]. Bears can act both as intermediate and as definitive hosts [72,73] due to their varied dietary habits: as herbivores and omnivores, they can act as intermediate hosts for some *Sarcocystis* species, whereas, as carnivores, they act as definitive hosts for other species when they consume an animal bearing sarcocysts. Both intermediate and definitive hosts can develop a variety of clinical symptoms depending on the parasite’s species, the infective dose, and the host’s immune status [74]. Recent studies on *Sarcocystis*-associated diseases, i.e., hepatic sarcocystosis and encephalitis, include two fatalities of a grizzly cub and a juvenile American black bear [75,76], showcasing the danger that the parasite can pose for young bears. Higher parasite prevalence was reported in older studies, namely, American black bears (11%) [55] and European brown bears in Slovakia (15.38%) [12]. Regarding Greek wildlife, *Sarcocystis* spp. have been detected in the faeces of golden jackals, red foxes, and European wildcats (definitive hosts) and in muscle tissue of wild boars (intermediate hosts) [59,73,74].

Two species of *Eimeria* have been identified in American black bears in previous studies, i.e., *Eimeria albertensis* and *Eimeria borealis* [77]. *Eimeria* spp. have also been found in grizzly bears [78], brown bears in Europe [12,13], and spectacled bears (*Tremarctos ornatus*) [79], with no species identification. *Eimeria* spp. infection in brown bears seems to have a low prevalence in Croatia (1.1%) and Slovakia (7.69%), in accordance with the present study (0.4%) [12,13]. *Eimeria* spp. are usually host-specific and infect a great variety of animals [80]. In Greece, the parasite has been detected in hares at a very high prevalence (64.28%) [81], as well as in poultry [82] and other domestic animals [83]. Eimeriosis mainly affects young animals, causing mainly enteritis and diarrhoea [81,84]. The presence of *Eimeria* spp. oocysts in the bears’ faecal samples may reflect true parasitism; however, cases of pseudoparasitism resulting from the consumption of infected prey are also likely. Species identification by molecular tools is needed to determine if bears are enzootic hosts of the taxon.

At least two cestode species of the Taeniidae family have been identified in ursids in the Northern Hemisphere, i.e., *Taenia krabbei* [48] and, in more recent genetic studies in Alaska and Finland, the Holarctic species *Taenia arctos* [85,86,87]. Both species have cervids as intermediate hosts. In Greece, there are only two endemic cervid species, i.e., *Capreolus capreolus* and *Cervus elaphus*, but there are no data on the presence of *T. arctos* in the country. The low prevalence recorded for Taeniidae (0.2%) in the present study is consistent with findings from other countries, where taenids were found by necropsy, as in the case of Finland and Canada, with a prevalence of 1.9% and 7.7%, respectively [85,87], or by combined faecal and post mortem examination as in Transylvania, with a prevalence of 3.2% [14]. In general, faecal examination is not very sensitive for detecting cestode infection, as infected animals often shed only a few free eggs and mostly release eggs within mature proglottids [59]. Furthermore, the diet of bears in Greece, which is mostly non-carnivorous [88], may explain the low prevalence of Taeniidae infection in the examined animals [85].

Eggs of the pentastomid *Linguatulla serrata* were recorded in two samples. *Linguatulla serrata* is a cosmopolitan zoonotic parasite affecting both herbivores and carnivores. Recent studies in Europe [89,90] show the presence of the parasite in both grey wolves (*Canis lupus*) and domestic ruminants. Studies from Greece also show the presence of the parasite in dogs, small ruminants, hares, and a variety of hospitalised wild mammals [81,90,91,92,93]. The parasite uses herbivores as an intermediate host and is transmitted when final hosts (carnivores) consume infected meat or internal organs [94]. Brown bears in Greece frequently consume ruminants [88] and might get infected. This is the first record of the parasite in brown bears, to the best of current knowledge. 

Seasonal dynamics of brown bear parasites have been mentioned in several studies, both in North America [51,78,95] and in Europe [15,17,18,19], all reporting a seasonal high in parasite prevalence in autumn and a low in spring. The present results show a similar seasonal trend, with a peak in parasite prevalence in autumn (LH) and a low in early summer (MA). The seasonal trend is driven by the most prevalent species, *B. transfuga*, whose occurrence varied strongly across seasons. Spatial hotspots were detected primarily during EH and LH, whereas no statistically significant hotspots were observed during EM and MA, suggesting that increased host movement during these phases reduces localised environmental contamination and thus infection pressure. However, the lower sample size in EM (n = 134) may limit the model’s ability to detect subtle spatial structures during this period. Although the prevalence was higher during EH than EM, the model results indicated that this difference was largely associated with spatial clustering, highlighting the importance of location-specific processes during EH and LH.

*Dicrocoelium dendriticum* infections showed relatively low overall prevalence but clear spatial structuring. The probability of infection increased significantly in later years, although this effect was modest compared with spatial patterns. While no significant spatial structure was detected during EM or MA seasons, suggesting a more homogeneous distribution of infections during these periods, a strong and significant spatial clustering emerged in both EH and LH, indicating that transmission becomes more spatially aggregated during these seasons. This pattern likely reflects seasonal changes in host foraging behaviour or environmental exposure, leading to localised infection hotspots. 

*Dicrocoelium dendriticum* shows significant and consistent spatial clustering across both EH and LH, implying a more persistent hotspot trait throughout the hyperphagia phase. In contrast, hotspots of *B. transfuga* appear more strongly concentrated in LH, aligning with its pronounced seasonal increase in prevalence and suggesting a sharper, more temporally focused aggregation of infection risk. Although the spatial hotspot analyses were based on modelled prevalence rather than raw case counts, sampling intensity was uneven across the study area. Areas with lower sampling intensity may therefore be associated with greater uncertainty in predicted prevalence. Consequently, the identified hotspots should be interpreted as regions of elevated infection risk within the limits of the available sampling coverage.

*Uncinaria* spp. showed no seasonal trend or spatial structure in any season, suggesting that other factors may affect its prevalence.

Finally, all three parasites (*B. transfuga*, *D. dendriticum*, and *Uncinaria* spp.) exhibited significant differences in prevalence among study years. Such variation may be associated with several factors, including sampling variation, climatic variation, fluctuations in intermediate-host availability, host population density, and habitat change; however, these factors were not evaluated in the present study because the necessary data were unavailable.

There is no definitive explanation why this seasonal trend is observed in brown bears. Several studies hypothesise that bears manage to void their intestines of helminth parasites before hibernation and get reinfected in spring [19,42,48,95]. Still, there is no clear evidence of the mechanism involved in this event. According to the present results, faecal samples positive for parasites were collected in early spring, suggesting that parasitism persisted through the hibernation period. However, the present results may be biassed due to several factors. Bears in milder climates, such as Greece, may not fully hibernate [22], and as a result, parasites persist year-round, potentially leading to biassed seasonal prevalence in the area. Furthermore, the low sample count during late spring–early summer (ΜA) may have led to an underestimation of parasite prevalence. Quantitative analysis of bear faeces to assess the abundance and excretion intensity of *B. transfuga* eggs has only been conducted in two studies from Slovakia [19,42]. Both studies show a peak in autumn, consistent with the seasonal pattern observed in the present study, where intensity of parasitism showed a progressive seasonal shift, with predominantly low egg counts (<50 EPG) in the first season, followed by an increase in intermediate EPG classes, i.e., 50–100 and 100–250 EPG in MA and EH seasons, respectively. The highest infection intensities were recorded in LH, where a substantial number of samples scored >250 EPG, while cases of low intensity infections declined markedly. Mixed infections with two or more different taxa were present in all seasons. The seasonal trend we observed above also seems to hold true for polyparasitism. Samples positive for more than one parasite species were most frequently observed during early and late hyperphagia, with parasite diversity being lowest during emergence and the mating period. Polyparasitism, although common in wild animals, remains under-researched and may have cumulative effects on the host’s immune system [96]. 

Additional limitations beyond those discussed above should be considered when interpreting the results of this study. First, pseudoparasitism cannot be excluded for certain parasite taxa, particularly given the omnivorous diet of the brown bear. Second, morphological examination alone was insufficient to identify several parasitic elements at the species level; therefore, molecular analyses would be required to confirm their taxonomic identity. Furthermore, the available data were insufficient to fully explain the temporal patterns detected by the GAMs. Spatial predictions generated by GAMs are based on smoothed surfaces and do not account for fine-scale environmental variables, such as vegetation structure, soil moisture, and microclimatic conditions, which may influence parasite distribution.

Despite these limitations, this study has several notable strengths, including the largest dataset of brown bear faecal samples examined for endoparasites in Europe to date. To the best of the authors’ knowledge, this is also the first study to employ the Baermann technique, the gold-standard coprological method for lungworm detection, thereby providing robust estimates of lungworm prevalence in the brown bear population studied. The findings provide important insights into the composition of the species’ endoparasite fauna and the seasonal variation in several parasite taxa. Future studies should incorporate genetic identification of individual bears to reduce the risk of pseudoreplication and should be complemented by parasitological examinations of carcasses to obtain a more comprehensive assessment of parasite diversity.

## 5. Conclusions

Ten endoparasite taxa were identified by faecal examination of brown bears in Greece. The results of the present study are consistent with findings from studies on brown bears in both Europe and North America. The sample size of the present study allows for the drawing of reasonably robust conclusions regarding the species composition and prevalence of endoparasites in the brown bear population of the study area, as well as the health risks these parasites may pose to this species, an aspect that warrants consideration given its vulnerable conservation status. However, to date, there is no unequivocal evidence of the actual impact parasitoses have on the brown bear population in Greece. Furthermore, some of the identified parasites may also infect other sympatric wild and domestic animals; therefore, brown bears may be considered among the hosts that contribute to the maintenance and transmission of these parasites within the study area. A seasonal trend in *B*. *transfuga* infection was confirmed herein, a finding that could be of relevance for brown bears’ general fitness status throughout their activity season. Further research is required to fully elucidate the life cycle of *B. transfuga*, its zoonotic potential, and its impact on the species’ general health. Additional genetic analyses are needed to further identify the parasites and eliminate potential bias arising from repeated sampling of the same individuals. Finally, long-term, systematic monitoring of bear parasites is essential to detect emerging changes, improve understanding of transmission dynamics, assess potential impacts on population health, and clarify the ecological processes that shape parasite distribution in this species.

## Figures and Tables

**Table 1:** Parasite taxa and their prevalence in copromicroscopical examination of brown bears in Greece.

Parasite	No Positive/ Examined Samples	Prevalence (%)	95% Confidence Interval
*Baylisascaris tranfsuga*	367/918	39.98	36.91–43.12
*Uncinaria* spp.	166/918	18.09	15.74–20.71
*Dicrocoelium dendriticum*	132/918	14.38	12.24–16.85
*Crenosoma* spp.	51/195	26.15	19.9–32.2
*Eucoleus aerophilus*	19/918	2.07	1.33–3.20
*Sarcocystis* spp.	5/918	0.54	0.23–1.26
*Toxascaris leonina*	4/918	0.44	0.17–1.13
*Eimeria* spp.	4/918	0.44	0.17–1.13
*Linguatulla serrata*	2/918	0.22	0.06–0.80
Taeniidae	1/918	0.11	0.02–0.62

**Table 2:** Summary statistics of the GAM binomial model used to predict the prevalence of *Baylisascaris transfuga* according to the season, the year of study, and the sample location defined by the coordinates of the sample. EM: emergence season, MA: mating season, EH: early hyperphagia season, LH: late hyperphagia season.

**Parametric** **Coefficients**		**Estimate**	**SE**	**Odds Ratio**	**z-Value**	**Pr(>|z|)**	**Sig.**
Intercept		−2.55	0.52		−4.95	7.48 × 10^−7^	***
Season	MA	−1.64	1.03	0.27	−1.60	0.109112	
	EH	0.26	0.95	0.96	0.27	0.784762	
	LH	2.76	0.48	4.19	5.69	1.26 × 10^−8^	***
Year	2022	0.64	0.25	1.90	2.59	0.009526	**
	2024	1.02	0.27	2.78	3.72	0.000202	***
**Approximate Significance of Smooth Terms**			**edf**	**Ref.df**	**X^2^**	***p*** **-Value**	**Sig.**
te (Long, Lat): Season = EM			5.13	5.87	8.71	0.1661	
te (Long, Lat): Season = MA			10.67	12.36	10.89	0.5687	
te (Long, Lat): Season = EH			11.09	12.51	24.18	0.0247	*
te (Long, Lat): Season = LH			3.00	3.01	9.19	0.0268	*
R^2^(adj) = 0.267 Deviance explained = 25.3%, n = 859							

SE: standard error; edf: estimated degrees of freedom (smooth terms), Ref.df: reference degrees of freedom for tests, Pr: *p*-value. * *p* < 0.05, ** *p* < 0.01, *** *p* < 0.001.

**Table 3:** Summary statistics of the gam binomial model used to predict the prevalence of *Uncinaria* spp. infections by year of study and sample location, defined by sample coordinates. EM: emergence season, MA: mating season, EH: early hyperphagia season, LH: late hyperphagia season.

**Parametric Coefficients:**		**Estimate**	**SE**	**Odds Ratio**	**z-Value**	**Pr(>|z|)**	**Sig.**
Intercept		−1.36	0.27		−4.99	5.95 × 10^−7^	***
Year	2022	−0.25	0.27	0.78	−0.94	0.346366	
	2024	−1.02	0.31	0.36	−3.32	0.000891	***
**Approximate Significance of Smooth Terms:**			**edf**	**Ref.df**	**X^2^**	***p*** **-Value**	**Sig.**
te (Long, Lat): Season = EM			8.61	9.97	14.29	0.149	
te (Long, Lat): Season = MA			8.40	8.82	13.63	0.206	
te (Long, Lat): Season = EH			7.88	8.52	12.45	0.132	
te (Long, Lat): Season = LH			11.52	13.72	12.16	0.564	
R^2^(adj) = 0.07 Deviance explained = 11.9%, n = 859							

SE: standard error; edf: estimated degrees of freedom (smooth terms), Ref.df: reference degrees of freedom for tests, Pr: *p*-value. *** *p* < 0.001.

**Table 4:** Summary statistics of the gam binomial model used to predict the prevalence of *Dicrocoelium dendriticum*. infections by year of study and sample location, defined by sample coordinates. EM: emergence season, MA: mating season, EH: early hyperphagia season, LH: late hyperphagia season.

**Parametric Coefficients** **:**		**Estimate**	**SE**	**Odds Ratio**	**z-Value**	**Pr(>|z|)**	**Sig.**
Intercept		−5.64	1.30		−4.33	1.49 × 10^−5^	***
Year	2022	1.06	0.39	2.89	2.69	0.0072	**
	2024	0.92	0.42	2.51	2.18	0.029	*
**Approximate Significance of Smooth Terms:**			**edf**	**Ref.df**	**X^2^**	***p*** **-Value**	**Sig.**
te (Long, Lat): Season = EM			6.56	7.28	9.27	0.3017	
te (Long, Lat): Season = MA			6.14	7.05	9.82	0.2029	
te (Long, Lat): Season = EH			12.68	13.96	34.21	0.0018	**
te (Long, Lat): Season = LH			9.25	10.35	29.45	0.0014	**
R^2^(adj) = 0.14 Deviance explained = 21.1%, n = 859							

SE: standard error; edf: estimated degrees of freedom (smooth terms), Ref.df: reference degrees of freedom for tests, Pr: *p*-value. * *p* < 0.05, ** *p* < 0.01, *** *p* < 0.001.

## Data Availability

The raw data supporting the conclusions of this article will be made available by the authors on request.

## Abbreviations

The following abbreviations are used in this manuscript:

GAMs	Generalised Additive Models
GPS	Garmin Instinct Solar
EM	Emergence
MA	Mating Season
EH	Early Hyperphagia Season
LH	Late Hyperphagia Season
UBRE	Unbiassed Risk Estimator
REML	Restricted Maximum Likelihood
AUC	Area Under the Curve
